# Modeling non-linear changes in an urban setting: From pro-environmental affordances to responses in behavior, emissions and air quality

**DOI:** 10.1007/s13280-022-01827-8

**Published:** 2023-02-03

**Authors:** Mira Hulkkonen, Roope O. Kaaronen, Harri Kokkola, Tero Mielonen, Petri Clusius, Carlton Xavier, Heidi Hellén, Jarkko V. Niemi, Jussi Malila

**Affiliations:** 1 Nano and Molecular Systems Research Unit, University of Oulu, P.O. BOX 8000, 90014 Oulu, Finland; 2 Past Present Sustainability Research Unit, Faculty of Biological and Environmental Sciences, Helsinki Institute of Sustainability Science, University of Helsinki, Viikinkaari 1, P.O. BOX 65, 00014 Helsinki, Finland; 3 Atmospheric Research Centre of Eastern Finland, Finnish Meteorological Institute, 70211 Kuopio, Finland; 4 Faculty of Science, Institute for Atmospheric and Earth System Research (INAR), University of Helsinki, 00101 Helsinki, Finland; 5 Atmospheric Composition Research Unit, Finnish Meteorological Institute, 00101 Helsinki, Finland; 6 Helsinki Region Environmental Services Authority (HSY), 00066 Helsinki, Finland

**Keywords:** Agent-based model, Atmospheric processes, Emissions, Human behavior, Particulate pollution, Urban mobility

## Abstract

Interactions in urban environment were investigated using a multidisciplinary model combination, with focus on traffic, emissions and atmospheric particles. An agent-based model was applied to simulate the evolution of unsustainable human behavior (usage of combustion-based personal vehicles) as a function of pro-environmental affordances (opportunities for sustainable choices). Scenarios regarding changes in multi-pollutant emissions were derived, and the non-linear implications to atmospheric particles were simulated with a box model. Based on the results for a Nordic city, increasing pro-environmental affordances by 10%, 50% or 100% leads to emission reductions of 15%, 30% and 40% within 2 years. To reduce ambient particle mass, emissions from traffic should decrease by > 15%, while the lung deposited surface area decreases in all scenarios ($-\,23\%$, $-\,32\%$ and $-\,36\%$, correspondingly). The presented case is representative of one season, but the approach is generic and applicable to simulating a full year, given meteorological and pollution data that reflects seasonal variation. This work emphasizes the necessity to consider feedback mechanisms and non-linearities in both human behavior and atmospheric processes, when predicting the outcomes of changes in an urban system.

**Supplementary Information:**

The online version contains supplementary material available at 10.1007/s13280-022-01827-8.

## Introduction

Urban systems, cities, are inherently complex due to the variety of constituents that form them and the myriad of different interactions occurring in them. A change in one part of the system can have non-linear and unintended consequences affecting another part of the system (Batty 2013; Wilson 2014; West 2017). This is especially relevant for understanding the consequences of dynamic human behavior that produces emissions of various pollutants. For achieving many sustainability goals, such as climate change mitigation and the reduction of air pollution, cities have been identified as the key implementation arena (Angheloiu and Tennant 2020). In order to study possible ways to achieve these goals, complexity must be taken into consideration.

Urban air pollution, including fine particulate matter, is detrimental for human health. Inhaled particles are associated with cardiopulmonary diseases, lung cancer, cognitive dysfunction and premature mortality (Pope III et al. 2002; Silva et al. 2013; Cipriani et al. 2018; Lelieveld et al. 2020). The lung deposition and resulting physical effects of particles have been shown to depend on the size, number concentration and surface area of particles (Oberdörster et al. 2005; Alföldy et al. 2009), which highlights the importance of studying these different metrics. For anticipating changes in concentrations of atmospheric particles, it is necessary to understand the multiple different processes affecting them. Correspondingly, for leveraging the adoption of sustainable behaviors leading to emission reductions, it is important to understand the mechanisms from which the behaviors emerge. These mechanisms can be explored with the help of formal models to study coupled socio-environmental systems (Polhill et al. 2016). The urban boundary layer as the interface between the anthroposphere and atmosphere is an example of such a system. To understand the total outcomes of changes in one part of the system, the models that are used must capture non-linearities and feedback mechanisms in both human and atmospheric processes.

Human systems and their evolution have been studied with system dynamics models and agent-based models (ABM) that allow dynamic and adaptive behaviors, heterogeneous populations and collective behaviors arising from different simple interactions (Bonabeau 2002; Grimm et al. 2005). Examples of ABM applications are available from different fields such as ecology (Grimm et al. 2005), epidemiology (Roche et al. 2011; Hunter et al. 2017), energy (Shafie-khah and Catalão 2015; Kühnlenz and Nardelli 2017), economy (Deissenberg et al. 2008), and transport (Hager et al. 2015; Ziemke et al. 2019). In the study of scenarios related to traffic and related emissions, various approaches involving ABMs have been applied. Most studies have aimed at accurate simulation of the travel times and routes of individual vehicles based on origin-destination surveys, after which the simulation outputs have been modified to represent different scenarios related to e.g vehicle fleet or traffic volumes (Hatzopoulou et al. 2011; Hofer et al. 2018). Some studies have focused more on capturing behavioral strategies and choices of the agents: Kangur et al. (2017) developed an ABM to simulate different consumer needs and individual-level decision-making to study the adoption of electric vehicles and corresponding changes in emissions, whereas Maggi and Vallino (2021) approached urban commuting and different policies to make it more sustainable by applying an ABM to simulate the economic, psychological and social drivers of decision-making in traffic mode choice. All the above-described traffic flow and traffic mode choice simulations have been augmented by applying emission factors for e.g. carbon dioxide (CO$_{2}$) and particulate matter to analyze the evolution of pollution corresponding to different scenarios.

The emissions enter the atmospheric boundary layer, where different meteorological and physicochemical processes govern the dispersion, possible conversion and removal of emitted compounds (Stull 1988). Changing human behavior and related emissions thus has atmospheric implications, the scale of which can only be understood by taking those processes into account. The existence of a link between traffic and ambient particle concentrations in urban areas is evident. The hourly variation of particle concentrations in cities typically follows the variation in human activities with a morning peak hour and afternoon peak hour during weekdays, and a different concentration level and profile during weekends (e.g. Masiol et al. 2014; Rivas et al. 2020). Large scale evidence of the atmospheric impacts of changing human behavior has been obtained after the start of the COVID-19 pandemic in 2020, as many cities around the world enforced lockdowns, considerably altering human activities (Diffenbaugh et al. 2020; Schulte-Fischedick et al. 2021). Decreased mobility and subsequent emission reductions revealed that the impact of this altered behavior on urban air pollution is significant, but, especially for particulate pollution, the impact is not linear (Kroll et al. 2020).

Human behavior involving combustion, such as traffic, produces primary emissions of particles, but the overall air quality impact is not limited to the primary emissions. Instead, the formation of secondary aerosol particles via gas-to-particle conversion involves various gaseous precursors, the prevalence of which is affected by traffic emissions (Kanakidou et al. 2005; Gentner et al. 2012). Changes in traffic emissions also imply indirect changes in particle formation via different reaction chains, such as by affecting the atmospheric oxidative capacity. Altered particle concentration is accompanied by changes in coagulation and in the potential of aerosol particles to grow by condensation, which affect the size distribution of particles. Thus, altered local emissions influence the total ambient aerosol concentration and the *particle size distribution* (PSD) via various mechanisms, the partial effects of which can modify the particle population to different directions. For example, in some analyses of air pollution changes during COVID-19 related lockdowns in spring 2020, enhanced secondary particle formation was identified to offset the reduction in particle concentrations that was expected due to reduced primary emissions (Huang et al. 2020; Ciarelli et al. 2021).

The different aerosol dynamic processes combined yield a non-linear response in particle concentrations as a result of changes in anthropogenic emissions. Thunis et al. (2021) have modeled this with focus on secondary inorganic aerosols while considering different emission reduction scenarios. Their results indicated a slight increase in the concentrations of particle mass in certain areas of the Po Valley, Italy, when emission reductions were applied. Other existing scenario studies of changing human behavior and related emissions, however, focus only on primary particle emissions and their dispersion. So although traffic emission scenarios have been studied in detail, insufficient attention has been paid to their indirect impacts and connection to aerosol processes such as the formation of secondary particles.

This paper sets out to demonstrate a novel model combination to study changes in unsustainable human behavior (the use of combustion-based personal vehicles in an urban environment) and related emissions, and corresponding changes in the atmospheric concentrations of particles. In particular, we aim to address non-linear mechanisms and feedback loops in the evolution of both behaviors and particle concentrations. Considering scenarios, we highlight the concept of *pro-environmental affordances* (Kaaronen 2017) that refers to opportunities for sustainable behavior. The main objective of this study is thus to investigate changes in air pollution (atmospheric particles in particular) as a function of increasing affordances to choose sustainable mobility

The rest of the paper describes the measurement data and methods that were used (“Materials and methods” section), presents the results and related discussion (“Results” and “Discussion” sections), and draws concluding remarks (“Conclusions” section).

## Materials and methods

### Target city

Helsinki is the capital and the largest city of Finland with 0.6 million citizens and 1.1 million inhabitants in its metropolitan area (Eurostat 2021). The trends in population and greenhouse gas emissions from traffic in Helsinki metropolitan area during 2000–2020 are depicted in Fig. S1 of Supplementary Information. In 2019, 60% of the population and workplaces in the region resided in zones associated with good accessibility to sustainable mobility (HSL Helsinki Region Transport 2018). From year 2012 to 2018, the percentage of sustainable mobility (public transport, cycling, walking) in all trips increased from 61% to 65% (HSL Helsinki Region Transport 2019). This corresponded to a change in traffic-related greenhouse gas emissions from 1.3 t to 1.2 t CO$_{2}$-eq per person.

### Measurement sites and data

The focus in this study is on the measurement site *Mäkelänkatu* (60$^{\circ }$ 11$'$ 47$''$ N, 24$^{\circ }$ 57$'$ 08$''$ E, 32 m a.s.l.), which is an urban street canyon site located at the edge of the central urban area of Helsinki city. The street alongside the measurement station is aligned in the northwest-southeast direction, and the width of the street canyon is 42 m. The heights of the buildings next to the measurement station and on the other side of the street are 19 m and 16 m. The location and geometry of the site are shown in Figs. S2a and S2b of Supplementary Information. The site was selected, because it represents a typical busy street in the city, and has comprehensive measurements regarding atmospheric composition. Simultaneous measurements include particle size distribution (PSD), particle number concentration (PNC), particle mass (PM), nitrogen dioxide, nitrogen monoxide and ozone (measurements operated by the Helsinki Region Environmental Services Authority HSY), and volatile organic compounds (VOCs, measurements described by Saarikoski et al. (in prep. 2022). Five days from 2019 with high quality measurement data and 100% coverage for all compounds were selected for the study: August 28th, September 3rd, September 6th, September 7th, and September 10th. The measurements are introduced in Table 1. Prevailing wind direction during the period considered in this study was southwest (the distribution of wind direction is displayed in Fig. S2b). However, turbulent mixing caused by traffic has been identified as the most important factor for pollutant dispersion at this site (Kuuluvainen et al. 2018). Traffic density near the measurement site is around 28 000 vehicles per working day (Statistics of the City of Helsinki). On a weekday, the average distribution of traffic volumes is bimodal with distinct peaks during 7–8 a.m. and 3–5 p.m. Average level of PM$_{2.5}$ (mass of particulate matter with aerodynamic diameters below 2.5 $\upmu \hbox {m}$) measured at the Mäkelänkatu site during 2015–2020 was 7.1 $\upmu \hbox {g}$ m$^{-3}$ with standard deviation of 4.7 $\upmu \hbox {g}$ m$^{-3}$ .

**Table 1:** Air pollution data from the street canyon measurement site Mäkelänkatu

Measured variable	Instrument
PNC [cm$^{-3}$], PSD (*d*$_{p}$ = 6–800 nm)	DMPS (Vienna type DMA, Airmodus A20 CPC)
PM$_{2.5}$ [$\upmu \hbox {g}$ m$^{-3}$]	TEOM 1405
NO$_{2}$, NO [$\upmu \hbox {g}$ m$^{-3}$]	Horiba APNA-370
O$_{3}$ [$\upmu \hbox {g}$ m$^{-3}$]	Horiba APOA-370
VOCs [ng m$^{-3}$]	TD-GC-MS

We used meteorological data from an urban site, *Kaisaniemi*, located in central Helsinki (60.18$^{\circ }$ N, 24.94$^{\circ }$ E, 3 m a.s.l.) approximately 2 km south-west from the Mäkelänkatu site. The data included air temperature, wind speed, wind direction, relative humidity and sea level air pressure with 10-min resolution. Radiation data including direct irradiation, diffuse irradiation, global irradiation and longwave radiation were obtained from an urban background station *Kumpula* (60.20$^{\circ }$ N, 24.96$^{\circ }$ E, 24 m a.s.l.). Sulphur dioxide (SO$_{2}$) measurements were from the urban measurement station *Kallio 2* (60.19$^{\circ }$ N, 24.95$^{\circ }$ E, 21 m a.s.l.). Regional background concentrations of NO, NO$_{2}$ and O$_{3}$ were obtained from the measurement station *Luukki* (60.31$^{\circ }$ N, 24.68$^{\circ }$ E) located about 20 km north-west from Helsinki city center.

The key measurement locations are shown in Fig. S2a of Supplementary Information. Apart from the PNC, PSD and VOC measurements, all data are openly available through the portal of the Finnish Meteorological Institute (Honkola et al. 2013).

### Evolution of behaviors

To study possible changes in collective human behavior, we applied an ABM designed by Kaaronen and Strelkovskii (2020). The model was originally developed to study the cultural evolution of sustainable behaviors that emerge as a product of personal, environmental and social factors and interactions. It allows to study the development of pro-environmental and ’non-environmental’ behaviors as a function of time and so-called *pro-environmental affordances* (defined as opportunities for sustainable action afforded by the environment, e.g. infrastructure). In this study, pro-environmental and non-environmental behaviors refer to the use of sustainable and unsustainable modes of mobility, respectively. Kaaronen and Strelkovskii (2020) have conducted an empirical validation study of the model with data regarding the proportions of cycling and driving in Copenhagen. They demonstrated that it is possible to realistically simulate real-life evolution of behaviors with the model. An agent-based modeling approach is justified for this purpose, because the simulation of collective behaviors involves sets of heterogenous agents, agents with cognitive capabilities, and behaviors emerging from various interactions between agents and their environment, all of which are often claimed to be captured more realistically by ABMs than by equation-based models (Wilensky and Rand 2015; Helbing 2012). However, future work could also consider, test and validate other modeling approaches, such as system dynamics models or equation-based models, to predict the evolution of collective behaviors.

The model is based on the elaboration and expansion of a classic heuristic formula from social psychology (Eq. 1 by Lewin 1936), according to which human behavior (*B*) is a function (*f*) of the person (*P*) and their environment (*E*):

1 $$\begin{aligned} B=f(P,E). \end{aligned}$$

Kaaronen and Strelkovskii (2020) expanded Lewin’s equation to include five feedback loops and thus to construct a dynamic system that evolves over time. Those feedback loops or causal links include (1) ecological information in a physical and socio-cultural environment specifying affordances (or psychologically salient opportunities for behavior, see e.g. Kaaronen 2017; (2) personal states (e.g. habits, skills, intentions, and attitudes) affecting and shaping behavior; (3) behavior feeding back to and modulating the personal states of individuals through processes of learning and habituation (the process of gaining habits, see e.g. Rankin et al. 2009; (4) behavior altering and shaping the environment through so-called cultural niche construction (the construction of non-random biases on behavioral selection pressures, Odling-Smee et al. 2003); and (5) social network where all behaviors occur and which leads to the transmission of behaviors through processes such as teaching and copying. In the model, the behavior of agents is an emergent function of the five mechanisms: affordances, personal states, individual learning and habituation, niche construction, and social learning.

The model comprises a pre-determined number of agents moving in a landscape of affordances. The agents encounter either pro-environmental or non-environmental affordances and act upon them by behaving pro- or non-environmentally with pre-determined yet changing probabilities. Agent behavior affects the behavior of other agents in their social network through social learning and niche construction, defined as rates in the model (listed in Table 2). The interactions between agents (social learning), niche construction, individual learning and habituation all cause changes in the probability of an agent to select the sustainable alternative: The probability of a sustainable choice increases or decreases by the rates assigned to the different processes. The collective behavioral patterns then emerge from the behaviors of agents that evolve as a result of the above-described feedback loops.

We applied the model so that the landscape of affordances was considered to represent people’s opportunities to select sustainable modes for mobility, i.e. public transport or active mobility, cycling or walking. In this context, we interpreted affordances to include physical infrastructure such as public transport and maintained cycling routes, accessibility (such as range and density of bus routes in relation to people’s mobility needs), cost, satisfaction (people’s perceived ease and convenience to select and use the sustainable modes), and availability of information regarding possibilities and their consequences.

Precise definition of several parameters applied in the model is not possible, as is typical for social system models (e.g. Smaldino 2017). In simulating complex systems, initial conditions can cause comparatively large variance in the end results (e.g. Muelder and Filatova 2018). This issue has been addressed by conducting numerous sensitivity tests with varying parameters. For local one-factor-at-a-time and global sensitivity tests of the model applied in this study, we refer to the Supplementary Information of the model description by Kaaronen and Strelkovskii (2020).

**Table 2:** Variable model parameters, their descriptions, and values applied in this study with underlying theory and/or reasoning

Model parameter	Description	Applied value	Reasoning
number of agents	Total number of agents in the model. One generation	300	The model is robust against the number of agents. Sensitivity tests indicate that increasing the number of agents further does not produce significant differences in the distribution of behaviors
pro-amount	Initial proportion of pro-environmental affordances in the system	0.45	A product of (1) the proportion of inhabitants in Helsinki living and moving in the zone defined to have “good accessibility to sustainable mobility” (60%, HSL Helsinki Region Transport 2018) and (2) stated satisfaction to service (76% for public transport, BEST 2018)
initial-pro	The initial pro-environmental personal state, which defines the probability to interact with pro-environmental affordances when encountered	0.65	The proportion of trips with sustainable modes of mobility in Helsinki in 2018 (HSL Helsinki Region Transport 2019). The development from 0.61 to 0.65 in the course of 6 years between 2012–2018 was used for validating the total parameter setup
initial-non	The initial non-environmental personal state, which defines the probability to interact with non-environmental affordances when encountered	0.35	The proportion of trips with unsustainable modes of mobility (personal vehicles) in 2018
construct-pro	The rate of pro-environmental niche construction	5	Default value derived based on sensitivity analysis by Kaaronen and Strelkovskii (2020)
construct-non	The rate of non-environmental niche construction	0	Default value derived based on sensitivity analysis by Kaaronen and Strelkovskii (2020)
social-learning	The rate of social transmission of behavior	$7 \times 10^{-5}$	Default value derived based on sensitivity analysis by Kaaronen and Strelkovskii (2020)
asocial-learning	The rate of individual learning and habituation	$5 \times 10^{-5}$	Default value derived based on sensitivity analysis by Kaaronen and Strelkovskii (2020)
network-param	*m0* in the Klemm–Eguíluz model. Defines the initial complete graph in the algorithm generating the network	5	Default value derived based on sensitivity analysis by Kaaronen and Strelkovskii (2020)
mu	${\mu }$ in the Klemm–Eguíluz model. Defines the probability of connecting with low degree nodes, and alters the clustering coefficient of the network	0.9	Default value derived based on sensitivity analysis by Kaaronen and Strelkovskii (2020)

The model with parameters from Table 2 was run for 100 iterations. In each iteration, we let the model run for up to 10 000 time steps, but monitored the status at intermediate steps 365, 730, 1095 and 3650 to quantify the evolved behaviors in short, medium and long term. One time step was regarded to represent one day. To create the scenarios representing increased pro-environmental affordances, we varied the parameter pro-amount from 0.495 (a 10% increase to the initial value) to 0.9 (a 100% increase).

The model has been implemented using NetLogo language and environment version 6.2.0 (Wilensky and Rand 2015). A full model description with the ODD (Overview, Design concepts, Details) protocol, pseudocode and UML diagram has been presented in the article by Kaaronen and Strelkovskii (2020) and its supplementary information. The model itself is available at 10.25937/z8x6-2v73.

As a result from the model runs, we obtained a percentage that represents change in the amount of non-environmental behavior (mobility with combustion-based personal vehicles) due to shifting to pro-environmental behavior (to active mobility or public transport). The change percentage is defined as

2 $$c_{{{\text{non}}}} = ({\text{non-env}} - {\text{initial-non}}){\text{/initial-non}} \times 100\%$$

with *initial-non* denoting the initial proportion of non-environmental behavior and *non-env* being the proportion of non-environmental behavior (at chosen time point) after modeled evolution of behaviors.

### From behavior to emissions

Direct emissions of different compounds were assumed to decrease in direct proportion to the change in behavior and corresponding changes in vehicle volumes: by the amount $c_{{{\text{non}}}}$ as determined in Eq. (2). When the use of a combustion-based personal vehicle is substituted with some sustainable mode of travel, we assume that the traffic emissions linked to the behavior of the agent decrease by 100%.

### From emissions to atmospheric concentrations

#### Nitrogen compounds

To deduce the fraction of traffic-originated primary NO$_{2}$ from the ambient concentration, we applied the *total oxidant method* proposed by Clapp and Jenkin (2001) and Carslaw and Beevers (2004), further developed by Keuken et al. (2009) and applied e.g. by Anttila et al. (2011). According to Clapp and Jenkin (2001), the mixing ratio of the total oxidant O$_{x}$ (i.e. the sum of O$_{3}$ and NO$_{2}$ mixing ratios) at an urban site can be approximated to be constituted by a regional background contribution that is NO$_{x}$ independent and a local NO$_{x}$-dependent contribution that correlates with local primary emissions. This is expressed as

3 $$\begin{aligned} {[}\text {O}_{x}]_\text {local}=a[\text {NO}_{x}]_\text {local}+b, \end{aligned}$$

where the mixing ratios of O$_{x}$ and NO$_{x}$ form a linear regression with regression parameters *a* and *b*. The slope *a* gives an estimate of the relative contribution of primary NO$_{2}$ emissions to the NO$_{x}$ mixing ratio. The subscript ’local’ refers to an observed mixing ratio with the effect of background concentration removed by calculating the difference between measurements in a street canyon (here, the Mäkelänkatu site) and at a background station (here, Luukki). After obtaining *a* from the least-square regression of measurement data according to Eq. (3), we applied it to construct the first term in NO$_{2}$ concentration budget with reference to Keuken et al. (2009). At an urban measurement site and at a moment *i*, the measured total NO$_{2}$ mixing ratio consists of a fraction from primary NO$_{2}$ emissions, conversion resulting from reactions between NO and O$_{3}$ and regional background:

4 $$\begin{aligned} {[}\text {NO}_{2}]_{\text {urban},i}= & {} [\text {NO}_{2}]_{\text {primary},i}\nonumber \\{} & {} +[\text {NO}_{2}]_{\text {conversion},i}+[\text {NO}_{2}]_{\text {background},i}, \end{aligned}$$

where

5 $$\begin{aligned} {[}\text {NO}_{2}]_{\text {primary},i}=a[\text {NO}_{x}]_{\text {local},i} \end{aligned}$$

Based on this approach, [NO$_{2}$]$_{\text {primary},i}$ as a fraction of measured [NO$_{2}$]$_{\text {urban},i}$ was calculated to be 9–21% with hourly variation. To create the scenario with changed behavior and correspondingly changed emissions, we modified the [NO$_{2}$]$_{\text {primary}}$ accordingly by multiplying it with $(1-c_{non}/100)$.

#### Volatile organic compounds

To derive the fraction of traffic-originated VOCs from the ambient concentrations, we based the analysis on the work by Hellén et al. (2003) focusing on measured non-methane hydrocarbons (NMHCs) in Helsinki. Using a chemical mass balance receptor model and a multivariate receptor model *Unmix* they have determined seasonal source contributions for measured C$_{2}$–C$_{10}$ hydrocarbon concentrations. For details of the measurements and applied methods, we refer to Hellén et al. (2003) and references within. Based on data collected in 2001 and the receptor models, Hellén et al. (2003) have determined the total contribution from traffic (liquid gasoline, gasoline vapor, gasoline exhaust and diesel exhaust) to the measured mass of NMHCs to be 57.3% in August. The fraction of NMHCs advected by long-range transported air masses was 37%. The determined source profiles were associated with a 15% uncertainty.

To create the scenario with changed behavior and correspondingly changed emissions, we multiplied the fraction in VOC concentrations that was assumed to be traffic-originated ($0.57\cdot$[VOC$_{n}$] for each traffic-originated VOC species *n*) by $(1-c_{\text{non}}/100)$.

#### Particles

The fraction of ambient particle concentration attributable to primary particle emissions varies with particle size. The fraction is also different when particle mass or particle number concentrations are considered. In the exhaust of vehicles, primary particles consist of soot particles in the size range of 30–500 nm (Kittelson 1998) and solid core particles with diameters below 15 nm (Rönkkö et al. 2017). Before getting diluted in the air, the exhaust has a very high temperature and contains different compounds (e.g. VOCs, sulphuric acid) in gaseous form. As the exhaust is emitted into the air, those compounds either nucleate or condense thus becoming part of the particle phase (Morawska et al. 2008). Rönkkö et al. (2017) have named them *delayed primary aerosols*. Primary and delayed primary particle emissions are important constituents of air quality near the emission source.

Typically, the PSD in engine exhaust is dominated by nucleation mode and Aitken mode particles (diameters < 10 nm and in the range 10–100 nm, correspondingly) for particle number (with more than 90% of emitted particles), and by accumulation mode particles (diameters in the range 100 nm–1 $\upmu \hbox {m}$) for particle mass (Kittelson 1998; Morawska et al. 2008). Rivas et al. (2020) have performed a seasonal source apportionment analysis for particle number size distribution in four European cities, including Helsinki. They identified fractions of total particle number concentrations attributable to traffic. For the Mäkelänkatu site, the relative contributions of different sources were identical in the annual average and autumn distributions: 18% from “traffic nucleation”, 37% from “fresh traffic” and 27% labeled as “urban”, which includes mostly delayed traffic-originated but possibly also other anthropogenic sources such as biomass burning (especially during cold periods). The total particle number concentration attributable to traffic was 12 603 ${{\hbox {cm}}^{-3}}$ corresponding to 82% of the total ambient number concentration. The traffic-originated fractions of total number concentration were converted into fractions specific for each particle size range. These are presented in Table 3.

For concentrations of sub-3 nm particles, Okuljar et al. (2021) have defined the relative contribution of traffic to be 47% in daytime, 65% in nighttime and 54% during the full measurement campaign at the Mäkelänkatu site in May–June. They observed that sub-3 nm particles at the background station were mainly linked to SO$_{2}$ and sulphuric acid concentrations, whereas at the street canyon site the particle concentrations were more correlated with compounds attributable to traffic (black carbon, NO$_{x}$, CO$_{2}$). According to measurement results from the same site by Hietikko et al. (2018), the daily profile of sub-7 nm particles follows the diurnal variation of traffic volumes. These results further emphasize the connection between traffic emissions and particle number concentrations at the site considered in this study.

To create the scenario with changed behavior and correspondingly changed traffic emissions, we modified the measured particle number concentrations by multiplying the size-specific fractions that were assumed to be traffic-originated by $(1-c_{\text{non}}/100)$. This, however, does not yet represent the new particle population, because it does not include the effects of particle formation, particle coagulation and condensation that also change with changed emissions and further shape the size distribution of particles. These dynamic processes were modeled as described in “Aerosol dynamic processes” section.

**Table 3:** Average traffic-originated fractions of size-dependent particle number concentrations for the urban street canyon site

Particle size (nm)	Fraction (%)	Reference
< 3	54	Okuljar et al. (2021)
3–10	68	Rivas et al. (2020)
10–25	81	Rivas et al. (2020)
25–250	73	Rivas et al. (2020)

Number concentrations were converted into mass concentrations based on the PSD and assuming spherical particles with density of 1000 kg m$^{-3}$. The conversion from number concentrations to *lung deposited surface area* (LDSA) considering alveolar deposition was performed with the ICRP Human Respiratory Tract Model (ICRP 1994). The LDSA is a relevant metric for the negative health effects of particles (Oberdörster et al. 2005).

### Aerosol dynamic processes

Apart from emissions, the time evolution of aerosol particles (their amount and size distribution) is governed by different dynamic processes: condensation growth involving existing particles and various vapors, coagulation among particles of different sizes, the formation of new particles via gas-to-particle conversion, and the removal of particles by wet and dry deposition. Simulating these processes requires numerous physical mechanisms and chemical reaction pathways to be taken into account. For this, a combination of models was used.

#### ARCA box model

The Atmospherically Relevant Chemistry and Aerosol box model (ARCA box) is a combination of existing models MALTE-box (Xavier et al. 2019), ADCHAM (Roldin et al. 2014) and ADiC (Pichelstorfer and Hofmann 2015) packaged by Clusius et al. (2022). A full description of ARCA box is provided by Clusius et al. (2022) and in an online documentation (ARCA 2022). Model version 1.1.2 was used in this study.

The ARCA box uses *Kinetic PreProcessor* (Damian et al. 2002), which solves the time evolution of gaseous chemical species by generating a system of coupled ordinary differential equations. The chemical reactions involving several organic compounds are from an online collection *The Master Chemical Mechanism*, MCM v.3.3.1 (Jenkin et al. 1997; Saunders et al. 2003). The photolysis rate of photochemically active species is calculated using the actinic flux derived from measured short wave radiation data. In the representation of PSD, the particles are divided into bins referred to with the diameter of the center of the bin. It is assumed that all particles in a bin have the same chemical composition, and all particles in the system are spherical with the same bulk density.

The formation of new particles is modeled with the explicit method *Atmospheric Cluster Dynamics Code* (ACDC, McGrath et al. 2012; Olenius et al. 2013) and a parametrization from Roldin et al. (2019). The parametrization is used for modeling clustering that involves sulfuric acid and organic compounds, whereas the ACDC simulates the formation of stable clusters for two-component systems of sulfuric acid and ammonia, and sulfuric acid and dimethylamine. The growth of particles by the condensation of organic vapors and sulfuric acid is calculated based on the saturation vapor pressures and concentrations of the compounds. The condensation mechanism is based on the Analytical Predictor for Condensation by Jacobson (2002). Coagulation, i.e. the process of particles in random motion colliding and agglomerating, is a rapid sink for small particles. It slowly increases the diameter of larger particles thus reshaping the PSD but not affecting total mass. In ARCA box, simulating Brownian coagulation is based on the discrete coagulation equation including number concentrations and coagulation coefficients (Seinfeld and Pandis 2016). Collisions between particles due to other reasons than Brownian motion, such as gravitational settling or wind shear, are not considered. Particles are removed from the air by wet and dry deposition. In this study, we did not consider either of these processes.

The input data for the ARCA box included all the data introduced in “Measurement sites and data” section, excluding data from the remote background station. Observations of total shortwave radiation, other meteorological variables, and concentrations of NO, NO$_{2}$, SO$_{2}$, CO, O$_{3}$ and VOCs are given as input for the model. Out of the measured VOC compounds, benzene, toluene, isoprene, $\alpha$-pinene, $\beta$-pinene, limonene, *o*-xylene, *m*-xylene, *p*-xylene, 1,2,4-trimethylbenzene, 1,3,5-trimethylbenzene and styrene were used. The measured PSD was used for initializing the model in the beginning of each run, and for background particle population. The measured PSD was extrapolated to cover sizes 1.4–6 nm based on measurements from the same site and similar meteorological conditions (temperature, air pressure, wind) in May 2018 as presented by Lintusaari (2019). The extrapolation was done by multiplying the concentrations of the smallest measured particles (6–9 nm) by factors that were determined based on ratios of concentrations for particle sizes 1.4–6 nm and 6–9 nm in the distributions reported by Lintusaari (2019). Variation in the shape of the distribution within a day was taken into account. As parametric input for ARCA, we used $J=3$ cm$^{3}$s$^{-1}$ for the particle formation rate (based on measurements in Helsinki by Hussein et al. 2008) and [NH$_{3}$] = $10^9$ cm$^{-3}$ for ammonia.

**Fig. 1:**
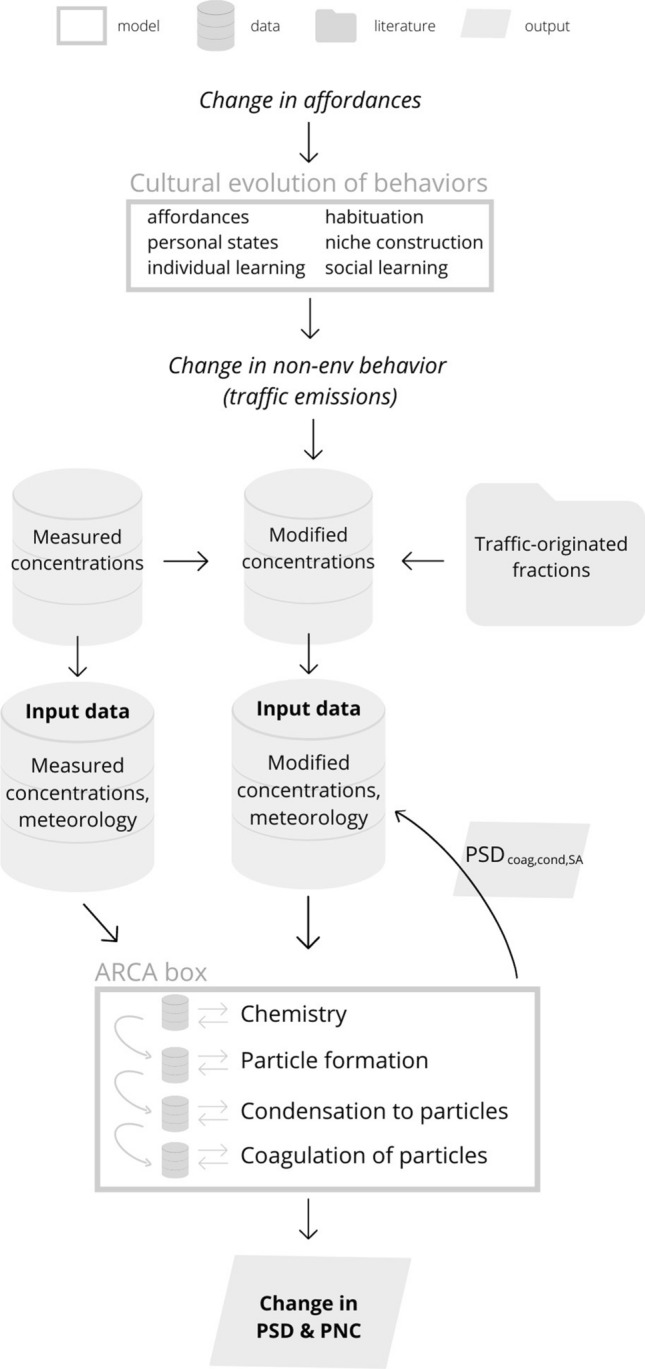
A schematic illustration of the full analysis flow starting with changes in affordances (that are input to the model simulating the cultural evolution of behaviors) and leading to the outcome (ambient particle size distribution and number concentration) of the atmospheric processes included in the ARCA box model

A schematic illustration of the full analysis flow including the steps described in “Evolution of behaviors”–“Aerosol dynamic processes” sections is presented in Fig. 1.

#### Change in PSD

To characterize the overall changes in particle size distribution, we considered changes in particles from primary emissions (as described in “Particles” section) and changes in aerosol dynamic processes (as described in “Aerosol dynamic processes” section) due to changes in the concentrations of both particles and gaseous compounds. To achieve the end result, i.e. the overall change in particle concentration and size distribution, we performed the following steps. Step 1: We ran the ARCA box model with the original measured particle population in the background. Step 2: we ran the ARCA box model initialized with modified (1−c) particle concentrations but without background particles, which yielded PSD$_\textrm{coag,cond,SA}$. Step 3: We re-modified the measured particle concentrations by calculating a weighted average:

6 $$\begin{aligned} PSD_\mathrm {input_{final}}=0.4 \cdot PSD_\textrm{coag,cond,SA}+0.6 \cdot PSD_{1-c}. \end{aligned}$$

Step 4: We ran the ARCA box model with PSD$_\mathrm {input_{final}}$ as background particle population. The final relative change in PSD was then obtained by comparing the output of ARCA box model ran with the original data (Step 1) and the output of ARCA box model ran with PSD$_\mathrm {input_{final}}$ (Step 4).

The adoption of this approach was inspired by the way in which greenhouse gas concentrations are modeled in the representative concentration pathway scenarios by the IPCC (Van Vuuren et al. 2011). There, the purpose is to first obtain the change in natural processes as a response to changed emissions, and then to include this impact of different scenarios to the concentrations of greenhouse gases in the final model run.

## Results

### The modeled evolution of behaviors

With the model setup as specified in “Evolution of behaviors” section, we simulated the evolution of pro-environmental and non-environmental behaviors as a function of time and pro-environmental affordances. The proportion of non-environmental behavior was interpreted as directly proportional to traffic emissions, i.e. emissions from using combustion-based personal vehicles for urban mobility. The abundance of pro-environmental affordances in the system (*pro-amount*) was defined as a combination of inhabitants residing within the zone associated with good accessibility to sustainable mobility and the stated satisfaction to the service related to public transport and cycling. Its initial level, 0.45, was determined based on data for Helsinki city.

**Fig. 2:**
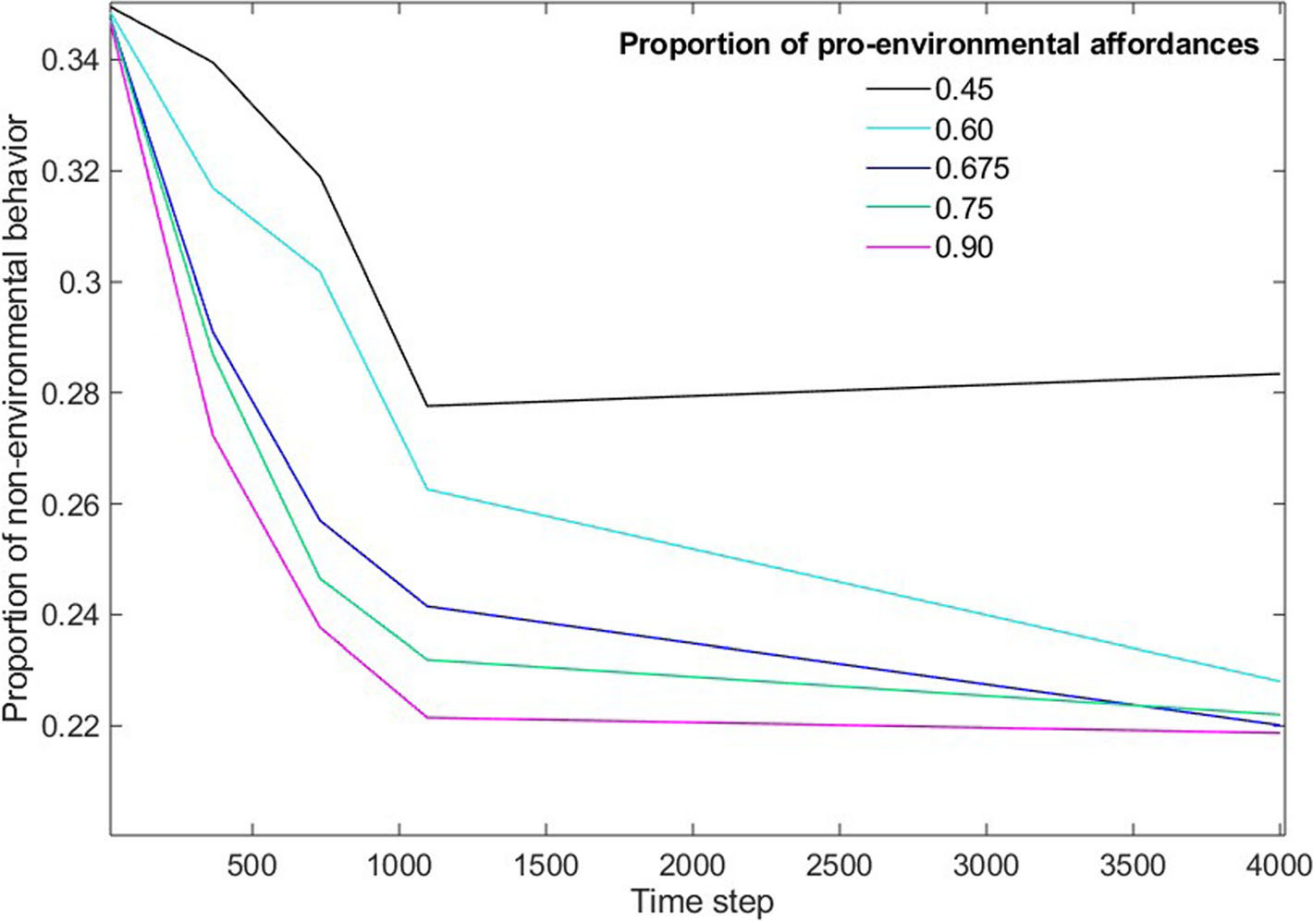
The evolution of non-environmental behaviors as a function of model time steps calculated with different values for the level of pro-environmental affordances. Average outcome of 100 iterations

The model was run for 100 iterations. The parameter pro-amount was varied between the initial value 0.45 and 0.9 corresponding to a $+$ 100% change. When pro-amount is below 0.5, as in the initial stage, the modeled behaviors may indicate such developments where the proportions of pro-environmental and non-environmental behaviors fluctuate strongly over time. In this situation, non-environmental behaviors may evolve to dominate over time, even if pro-environmental personal state has a higher initial value (*initial-pro* = 0.65) than non-environmental personal state. Figure 2 shows the average evolution of non-environmental behavior with different values for the parameter pro-amount, calculated based on 100 model runs for each case. In the Figure, the unpredictability of collective behaviors with pro-amount < 0.5 is visible, as the proportion of non-environmental behavior increases towards the end (time step 3650, corresponding to 10 years). With increasing values of pro-amount, the decline of non-environmental behavior becomes more rapid, but the curves converge over time. As is typical for cultural evolution (Mesoudi 2011), the adoption of new behaviors (which in this study means simultaneous decrease of opposite behaviors) follows an S-shaped cumulative distribution curve.

**Fig. 3:**
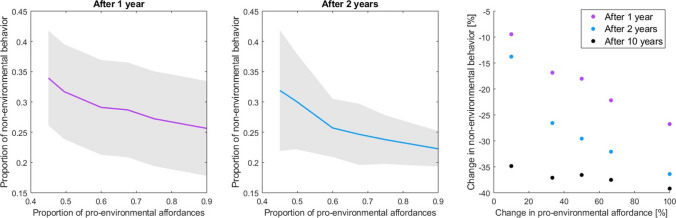
Change in non-environmental behavior against change in pro-environmental affordances. From the left: simulated status after 1 year (average of 100 model runs with ± 1 standard deviation), simulated status after 2 years, and percentage change in non-environmental behavior as a function of percentage change in pro-environmental affordance after 1, 2 and 10 years of simulated evolution

When the level of pro-environmental affordances increases in the system, selecting sustainable modes of mobility becomes more and more probable than the alternative. This is evident in the model results depicted in Fig. 3, which shows the change that occurs in the proportion of non-environmental behaviors as a function of pro-environmental affordances. With increasing pro-amount, the proportion of non-environmental behaviors decreases exponentially before settling to a new steady state. The increase in pro-environmental choices is linked to the mechanisms included in the model: Social learning and habituation cause a bias towards sustainable choices to diffuse in the social network as they alter the personal states of the agents, modify the environment through niche construction and thus trigger a positive feedback loop.

As was done by Kaaronen and Strelkovskii (2020) in their case study of Copenhagen, we interpreted the model time steps as days. We observed the state of evolving behaviors after 365, 730, 1095, and 3650 time steps, representing the state of the system 1 year, 2 years, 3 years and 10 years after the start of simulation (and after introducing the system to an increased level of pro-environmental affordances). The model parameters including the rate of learning and habituation were constant over time, although they could be expected to have some seasonal variation in real life. Within the time frame, a maximum change of − 40% (from 0.35 to 0.21) in non-environmental behavior was simulated. Increasing the affordances for pro-environmental behavior by 33% can lead to the maximum change within 10 years. A quicker change towards − 40% in non-environmental behavior requires that the pro-environmental affordances increase significantly, by up to 100% (from 0.45 to 0.9), which means that more than 90% of people would have good accessibility to *and* high satisfaction in sustainable mobility.

For modeling the atmospheric impacts as a function of changes in pro-environmental affordances, we focused on the simulation results at time step 730, corresponding to an evolution of 2 years. These results link the increase of pro-amount and the decrease of emissions as follows: + 10% in pro-amount corresponds to an emission change of − 15%, $+$ 50% to − 30% and $+$ 100% to − 40%.

### The modeled changes in aerosol dynamics

Percentage changes in behavior and emissions were converted into concentration changes of NO$_{x}$ compounds, VOCs and particles with the approaches specified in “From emissions to atmospheric concentrations” and “Aerosol dynamic processes” sections. The ARCA box model was then run with the original data and the modified data corresponding to changes in behavior. We validated the use of ARCA box model by making sure the simulation captures the initial particle distribution correctly.

#### Modeled changes in chemistry

The ARCA box model was used with the original data and the data representing changed emissions (− 40% as maximum change). Comparing the two, we observed the occurrence of changes in relevant chemical reactions and processes. The vapors contributing to cluster formation and condensation were H$_{2}$SO$_{4}$ and various highly oxygenated molecules (HOMs) produced by reactions involving VOCs. No significant differences in chemistry were observed when running the model with the original versus the $c=0.4$ scenario data. Noteworthy differences occurred in the concentrations of H$_{2}$SO$_{4}$ and HOMs: maximum daytime changes were approximately + 70% and − 50% correspondingly. The concentration of hydroxyl radical (OH) was slightly (< 10%) higher after the 40% emission reduction than with the original input data. These changes co-occurred with slightly enhanced particle formation rate, and enhanced particle growth by condensing vapors, when comparing the model output achieved with the $c=0.4$ scenario to the output with the original data. The enhanced particle growth was visually observable in the time evolution of the particle number size distribution (PNSD). For nitric acid HNO$_{3}$, we observed no differences between simulations done with the original data and the scenario. Differences would be expected in a simulation considering a more polluted urban environment (Kulmala et al. 2021). Based on the model results for Helsinki, the most significant chemistry-related changes occurred in the condensation growth of particles and in the PSD, which, in turn, produced further changes in coagulation and PNC (as described in the following section).

#### Modeled changes in particle population

In particle concentrations, we expected changes due to (1) changed primary emissions of particles, (2) changed concentrations of precursor compounds of secondary particles, and (3) changes in the dynamic aerosol processes including coagulation and condensation. In addition to changes in overall aerosol population, we expected changes in different particle size modes: nucleation mode (particle diameters below 10 nm), Aitken mode (10–100 nm) and accumulation mode (0.1–1 $\upmu \hbox {m}$). Nucleation mode particles contribute the most to the number concentration, whereas larger particles are important for total LDSA and mass.

**Fig. 4:**
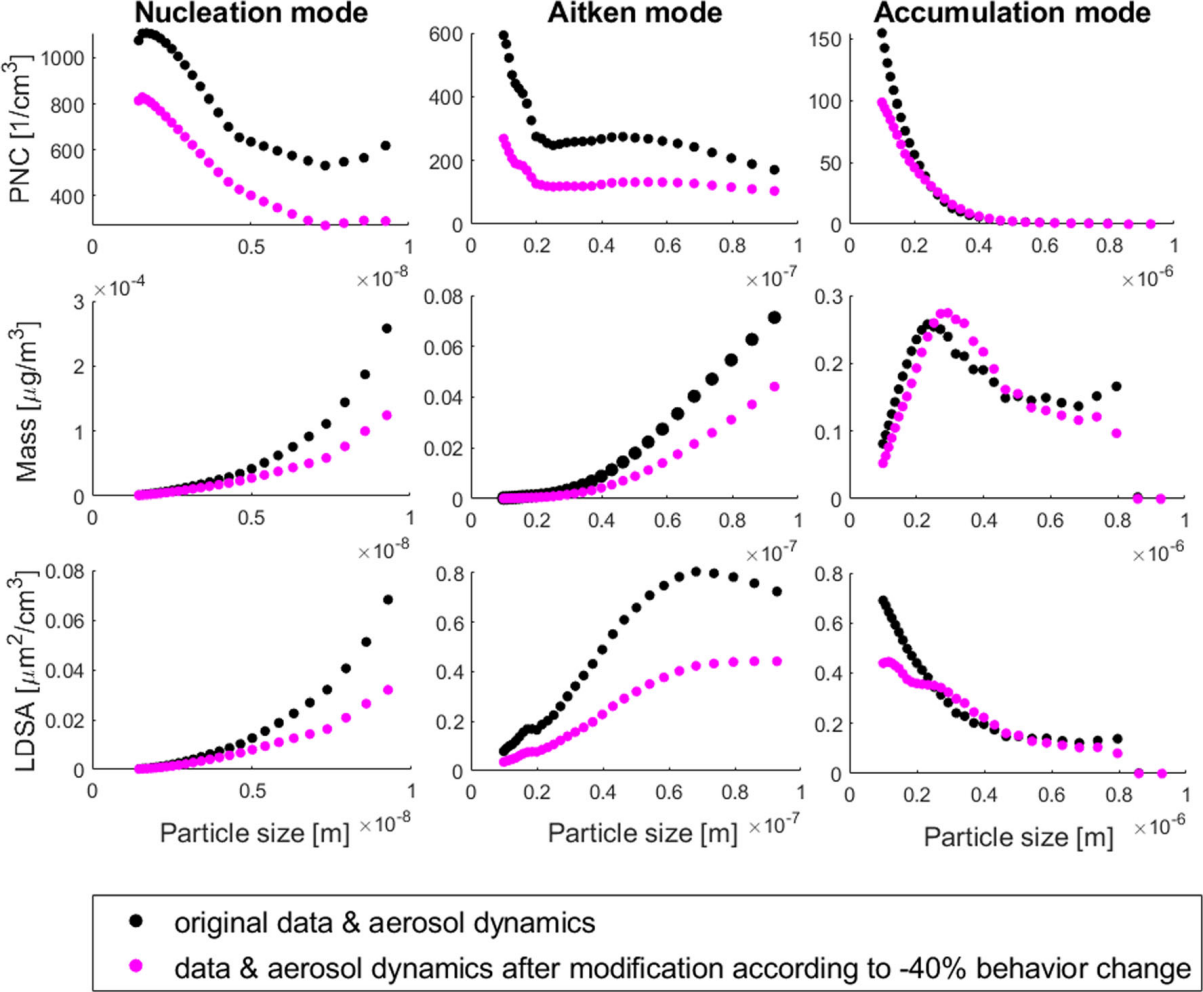
Total average (all modeled days) particle number concentration (PNC), particle mass concentration and lung deposited surface area (LDSA) for different particle size modes modeled with ARCA box model using the original data and the modified data corresponding to the maximum reduction of emissions ($c=0.4$)

Figure 4 shows the results of aerosol processes simulated with the ARCA box model using the original measurement data and the twice modified data (as specified in “Change in PSD” section) corresponding to a 40% decrease in emissions. Changes in particle number and mass concentrations, and in the LDSA are observable in all three size modes. The changed emissions did not change the concentrations linearly, but the aerosol processes produced different changes for particles in different size modes and for different metrics. In nucleation mode and Aitken mode, reduced emissions yielded a reduced level of PNC, mass concentration and LDSA. In accumulation mode, the model results indicated increased concentrations of particles with diameters > 0.2 $\upmu \hbox {m}$ when comparing the emission reduction scenario with the original situation. This effect was due to enhanced condensation growth, which “transfers” particles from the nucleation and Aitken modes to accumulation mode. The increased number concentrations in the accumulation mode are emphasized when considering particle mass.

**Fig. 5:**
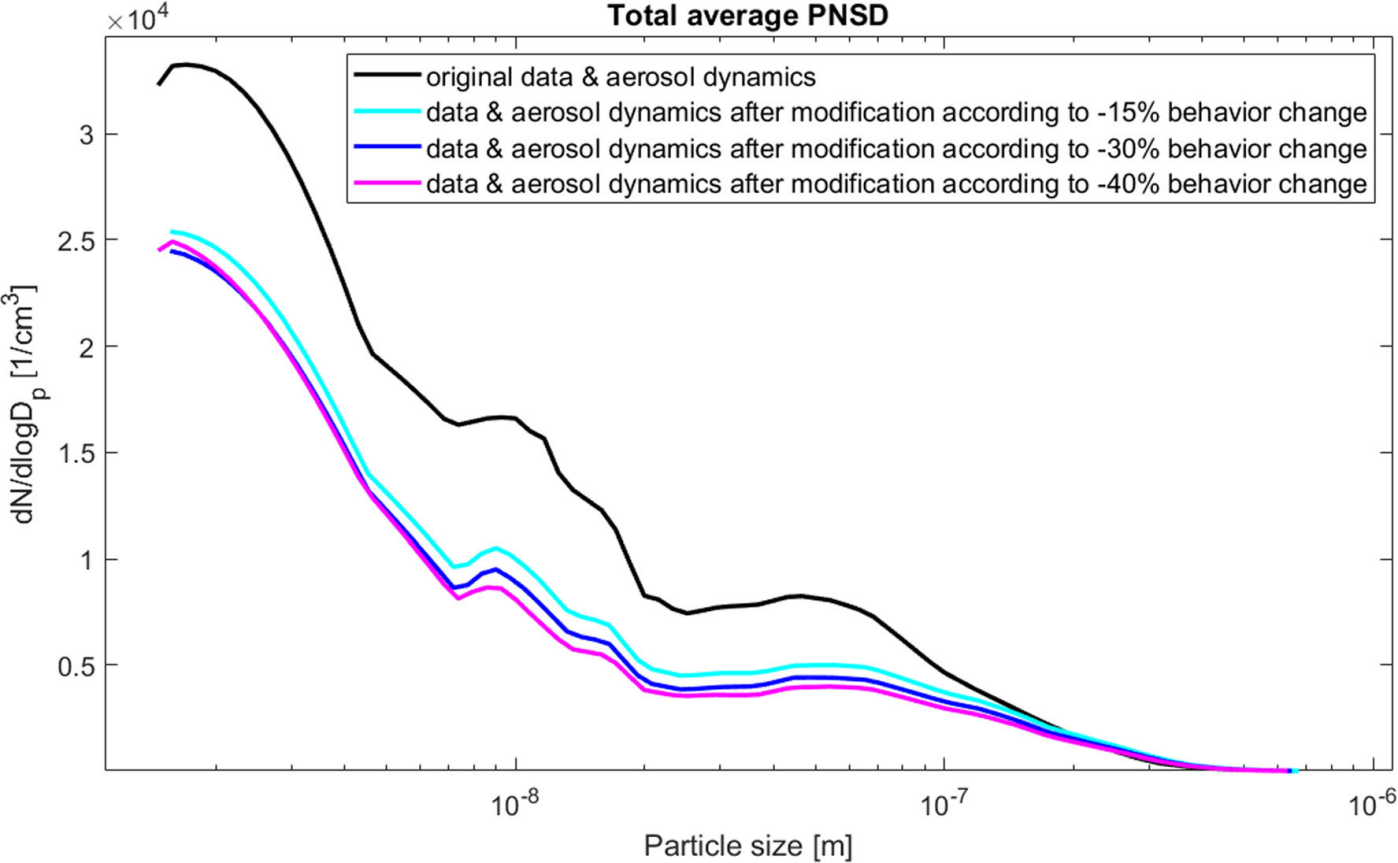
Total average of normalized particle number size distribution in the original situation and in the scenarios corresponding to 15%, 30% and 40% emission reduction

Total average PNSD in the original situation and for all scenarios is depicted in Fig. 5. The normalized particle distributions show that a significant change in number concentrations was modeled with all scenarios. Regarding particles with sizes > 0.2 $\upmu \hbox {m}$, all emission reduction scenarios produced increased number concentrations. The model results also indicated that the *coagulation sink* i.e. the loss rate of small particles due to scavenging by larger particles (Kulmala et al. 2001) decreased by − 25% on average when the emissions were reduced by 40%, but increased by $+\,1.85\%$ when emissions were reduced by 15%. The combination of enhanced condensation growth and enhanced coagulation amplified the decrease of PNC in the scenario with a 15% emission reduction.

**Fig. 6:**
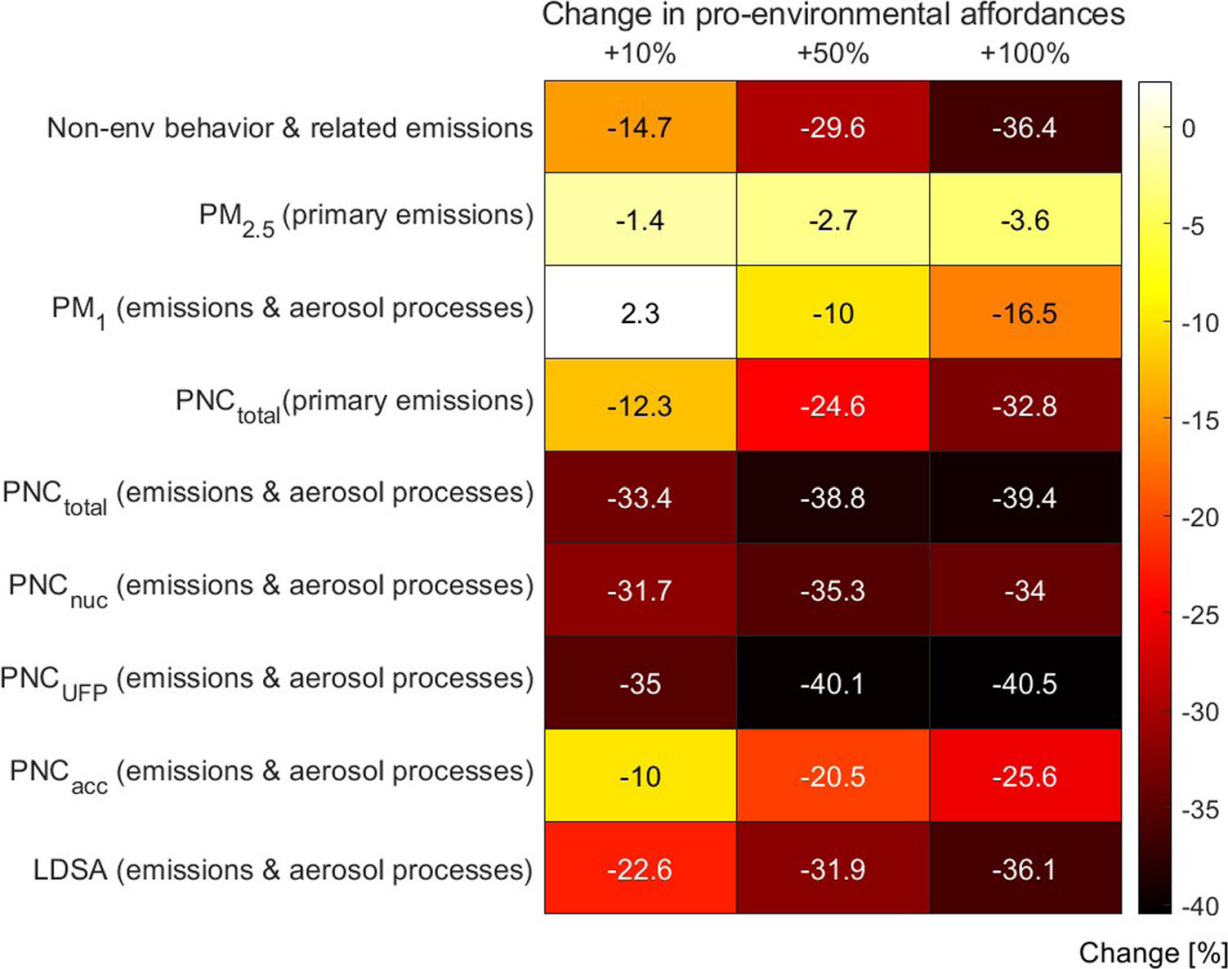
Percentage changes in non-environmental behavior & related emissions, particle mass (PM), particle number concentration (PNC: total, nucleation mode, ultrafine particles UFP, accumulation mode) and lung deposited surface area (LDSA) corresponding to changes in pro-environmental affordances

A *change matrix* in Fig. 6 shows the connection between changes in pro-environmental affordances and changes in emissions linked to non-environmental behavior (the use of combustion-based personal vehicles for mobility in an urban environment), in particle mass (PM), in particle number concentrations, and in the lung deposited surface area of particles. We specified the changes in PM and PNC so that the changes due to primary emissions and the combined changes due to emissions (PSD$_{1-c}$) and aerosol processes (PSD$_\text {coag,cond,SA}$) are shown separately. The columns indicate different levels of pro-environmental affordances compared to the baseline.

Considering decreased emissions only, the analysis yielded relatively small changes in total ambient PM$_{2.5}$ ($-\,1.4\%$, $-\,2.7\%$ and $-\,3.6\%$ for a 10%, 50% and 100% increase in pro-environmental affordances, correspondingly), while the changes in total ambient PNC were $-\,12.3\%$, $-\,24.6\%$ and $-\,32.8\%$, correspondingly. When taking both emissions and aerosol processes into account, ambient PM$_{1}$ decreased by up to 16.5% in the scenario with the largest emission reduction. Traffic emissions contribute more to particles with diameters below 1 $\upmu \hbox {m}$, whereas PM$_{2.5}$ in Helsinki is heavily affected by biogenic and aged aerosol particles, and long-range advection (Aarnio et al. 2016). For this reason, it was expected that decreased emissions affect PM$_{1}$ more than PM$_{2.5}$. However, the model results indicated that the enhanced growth of particles by condensing vapors in the emission reduction scenarios increased the particle concentrations in the accumulation mode, as is seen in Fig. 4, and this effect has a relatively larger significance for total mass than total number concentration. Regarding the scenario with 15% emission reduction, the modeled increase in accumulation mode concentrations overcompensated the reductions in the concentrations of smaller particles, which yielded an increase ($+$ 2.3%) in total PM$_{1}$. Based on this, a large enough reduction (> 15%) in multi-pollutant emissions from traffic would be required to reduce the level of PM$_{1}$ in Helsinki.

The decline in total PNC due to primary emissions only was in linear correlation with the emission reductions. When aerosol processes were included in the analysis, the relationship between emission reductions and changes in concentrations became non-linear. For the total particle number concentration dominated by ultrafine particles (UFP, particle diameters < 100 nm), a significant decrease of 33–39% was modeled with all emission reduction scenarios. Considering the negative health effects of atmospheric particles, toxicological studies have shown that PNC may not be as relevant a metric as LDSA (e.g. Oberdörster et al. 2005). The change in total LDSA considering alveolar deposition was a decrease in all scenarios ($-\,22.6\%$, $-\,31.9\%$, $-\,36.1\%$). The modeled absolute levels of LDSA were in line with measured levels in Helsinki (Kuuluvainen et al. 2016).

## Discussion

Uncertainties in our study are related to both data and model approaches.

The agents in the ABM model do not move in a simulated physical space, such as streets, but in a “landscape of affordances”. In real life, traffic as an emission source has strong spatial variability, which could be captured by a more traditional ABM, such as traffic flow simulation coupled with emission factors. Such approaches are dependent on detailed data from origin-destination surveys, and the models typically lack the mechanisms relevant for the cultural evolution of behaviors. We had high quality atmospheric measurement data and were able to lean on several earlier studies determining source contributions of different pollutants for our specific site. This enabled us to focus on the social and environmental processes, such as interactions, affecting urban mobility, instead of having to paint the picture of traffic as an emission source in Helsinki from scratch.

We modeled the evolution of mobility choices, but did not consider changes in technology, such as electrification of traffic and renewal of vehicle fleet, or in regulation, such as directives for fuel composition and particle emissions. These would affect traffic emissions even if no changes in the traffic mode choices of people occurred. Thus, the division to sustainable versus unsustainable mobility and the scenarios considered in this study form a simplified approach to traffic emissions.

The particle measurement data did not cover the smallest particles with diameters below 6 nm, which is why we extended the data down to 1.4 nm using previously published measurement results from the same site and similar conditions. This step proved to be necessary, because the results with particle measurement data starting at 6 nm were significantly different as demonstrated in Supplementary Information, Fig. S3 and Table S1. The model outcome was observed to be sensitive to the particle size range covered by the input data, and omitting the smallest (sub-6 nm) particles led to results that allow misleading conclusions.

The data was mainly from one measurement site and only covered 5 days from August-September, which does not enable wide generalization. Seasonal differences are very relevant for the behavior of people and the atmospheric composition in a Nordic city with significantly different conditions in winter and summer. Both behavior change (driven by e.g. the rate of learning and habituation) and aerosol processes (driven by atmospheric conditions and photochemistry) look different in different seasons. Regarding change towards more sustainable mobility, it is likely that the rate of learning and habituation is higher during pleasant summer conditions than in the winter. Correspondingly, the traffic-originated fractions of pollutant concentrations and the processes governing particle size distribution vary seasonally (Rivas et al. 2020; Virtanen et al. 2006; Karl et al. 2016). The presented modeling case with specific parameters and input data is representative of one season only. If the approach was applied for simulating full years, the parameters of the ABM, the traffic-originated fractions of pollutants and the meteorological data should include the seasonal variation.

The determination of traffic-originated fractions of pollutants was mostly based on earlier studies, the methods of which are associated with different uncertainties. The model for the evolution of human behaviors and the scenarios with a varying level of pro-environmental affordances include uncertainties that are unquantifiable in the absence of numerical data describing the different feedback mechanisms in social processes. The model setup for aerosol processes (ARCA box) includes some documented uncertainties. Not all possible chemical reactions are included in the model, such as the formation of highly oxygenated molecules from some compounds including ethene and pentane, which may be important constituents of anthropogenic VOCs especially in polluted cities (e.g. Tan et al. 2011). However, they are not expected to contribute significantly to the formation of particles, and in the case of a Nordic city with relatively low levels of pollutant concentrations, we assume that this is not a significant source of error. In general, there are still several open questions regarding the formation and growth of atmospheric particles (Yli-Juuti et al. 2020).

We performed calculations and model simulations for a specific measurement location in Helsinki, Finland. The analysis presented in this study was dependent on local measurement data and previous scientific studies determining contributions of traffic to ambient concentrations of different pollutants. For another urban environment with different conditions, the outcome would likely be different. It is expected that more drastic changes in the formation of secondary pollution would occur in a city with more atmospheric pollution, as the decreasing emissions could imply a larger increase in the oxidative capacity and accompanying changes in gas-to-particle partitioning. Also, the mixture of pollutants linked to traffic may be different in different cities depending on their vehicle fleet and fuel composition, and also meteorology. The emission factors of particles are not static but dependent on ambient conditions (e.g. Janhäll et al. 2012), and the dispersion of emissions is dependent on wind flow characteristics. Thus, the same behavior and changes in it would have different atmospheric impacts in different conditions. This is why the results from this study can not be used to derive universal conclusions regarding the connection between increasing pro-environmental affordances and changes in local air quality. Instead, we provided a case study analysis and offered a novel heuristic and model approach that enable studying that connection.

## Conclusions

Making sustainable mobility easier and more accessible could significantly affect the traffic mode choices of urban citizens, which would imply changes in the emissions of various pollutants. This is relevant for climate change mitigation and the prevention of air pollution such as particles associated with severe negative health impacts. Changes in an urban system do not typically happen linearly due to the various interactions between people, their activities and the environment. The ability to understand the possibilities to reduce air pollution requires that those interactions and causalities in both human behavior and in the atmosphere are considered, as was demonstrated in this study.

We investigated the connection between pro-environmental affordances, i.e. opportunities to select sustainable modes of mobility, and air pollution in a Nordic city. Special focus was placed on the ability to capture non-linear responses in both emitting behaviors and atmospheric processes. If the system has pro-environmental personal states (which means that some people make sustainable choices) and the affordances for pro-environmental behavior get increased, it may be expected that cultural evolution and self-organization in the system result in self-reinforcing sustainable behavior patterns. That, in turn, leads to emission reductions and changes in local air quality.

We modeled the cultural evolution of behaviors with realistic assumptions regarding socio-behavioral development, and linked that evolution to modelled consequences in particulate pollution. Based on simulation results, an improvement in pro-environmental affordances leads to a non-linear decrease in combustion-based mobility, which, in turn, yields decreased emissions of CO$_{2}$, particulate matter, NO$_{2}$ and VOCs. Already a 10% increase in pro-environmental affordances produced a reduction of 15% in emissions. The maximum modelled emission reduction was 40%, which was achieved within 2 years in a scenario where more than 90% of people would have good accessibility to and high satisfaction with sustainable mobility. The changes in the emissions of different pollutants affect the atmospheric composition and aerosol dynamics in a non-linear way, which we modeled with a box model covering relevant aerosol dynamic processes. Based on the model results, multi-pollutant emissions from traffic should be reduced by more than 15% to reduce the ambient PM$_{1}$ concentration. Considering particle number concentration, significant reductions of 35–40% in ultrafine particles were simulated with all scenarios corresponding to emission reductions of 15–40%. Also regarding the lung deposited surface area, which is possibly the most important metric describing the inverse health effects of particles, reductions were achieved with all emission reduction scenarios.

The modeling case presented in this study uses specific parameters and input data representative of one city in one season. The suggested approach, however, is generic and could be applied to simulating a full year of evolving behaviors and air pollution concentrations, given city-specific, meteorological and traffic-related pollution data that capture seasonal variation.

The pro-environmental affordances in the model of human behavior are related to infrastructure, but also include concepts such as psychological salience, capabilities, perception and satisfaction. Referring to Kaaronen and Strelkovskii (2020), a bicycle path will only afford bicycling for a person who knows how to cycle. Thus, this model does not enable recommending specific measures, but emphasizes the need for a holistic view when designing initiatives and measures for advancing sustainable urban mobility. The constituents of psychological salience and overall satisfaction with opportunities and services need to be understood to achieve realistic and city-specific model outcomes that enable policy recommendations.

Considering changes in collective behavior, a lag or delay after increasing the affordances for pro-environmental behavior is evident in the model results. And considering changes in atmospheric pollution, it is distinct that aerosol processes such as particle coagulation, condensation and the formation of new particles shape the outcome to an extent comparable to emissions and their dispersion. These aspects are relevant for the expectations regarding air pollution mitigation efforts and should be taken into account in policy making. Importantly, when designing air quality plans, a non-linear response in particle concentrations due to emission reductions should be expected, and that response is highly dependent on local conditions, atmospheric chemistry and the ambient concentrations of various compounds involved in aerosol dynamic processes.

The results of this study emphasize the necessity to consider various processes and feedback mechanisms in a holistic manner to be able to predict changes in an urban system, in human behavior and in air quality. For a complete picture of the link between human behavior and atmospheric composition, more research, measurements and evidence that enable formal quantification of both social and atmospheric processes are needed.

## Supplementary Information

Below is the link to the electronic supplementary material.

Supplementary file 1 (pdf 512 KB)
