# Supplementary material for: Modeling non-linear changes in an urban setting: From pro-environmental affordances to responses in behavior, emissions and air quality
Source: Ambio. 2023 Feb 3;52(5):976–94. doi: 10.1007/s13280-022-01827-8 (PMC9897621; doi:10.1007/s13280-022-01827-8)
Supplement: Supplementary file 1 — (pdf 512 KB) [file 13280_2022_1827_MOESM1_ESM.pdf]

Ambio

Supplementary Information
This supplementary information has not been peer-reviewed.

Title: **Modeling non-linear changes in an urban setting: From pro-environmental affordances to responses in behavior, emissions and air quality**

Authors: Mira Hulkkonen, Roope O. Kaaronen, Harri Kokkola, Tero Mielonen, Petri Clusius, Carlton Xavier, Heidi Hellén, Jarkko V. Niemi, Jussi Malila

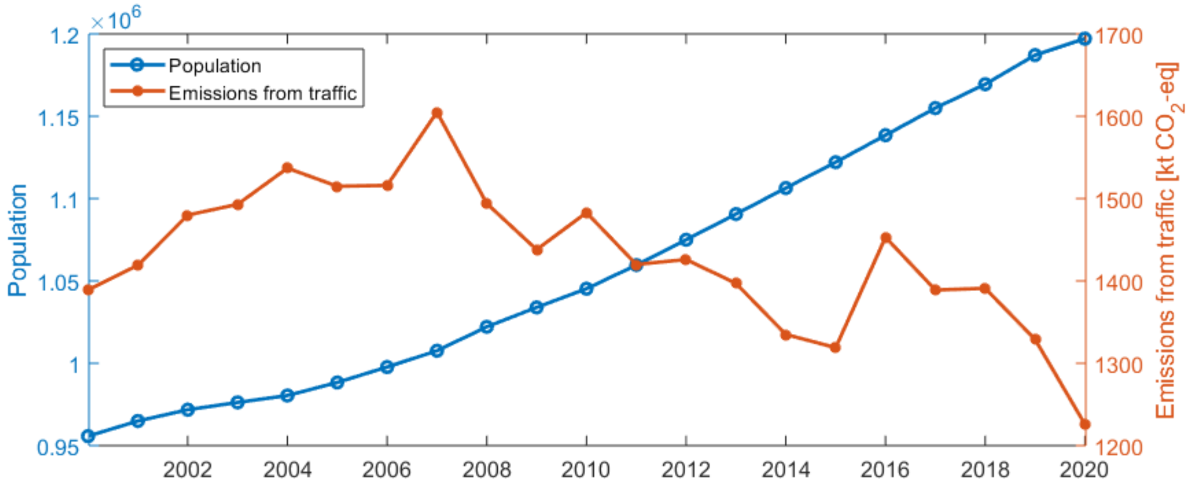

Figure S1: Population and greenhouse gas emissions (as kt CO_2-eq) from traffic in Helsinki metropolitan area during 2000–2020. Data: Statistics Finland (2021)[1] and Helsinki Region Environmental Services Authority (2021)[2].

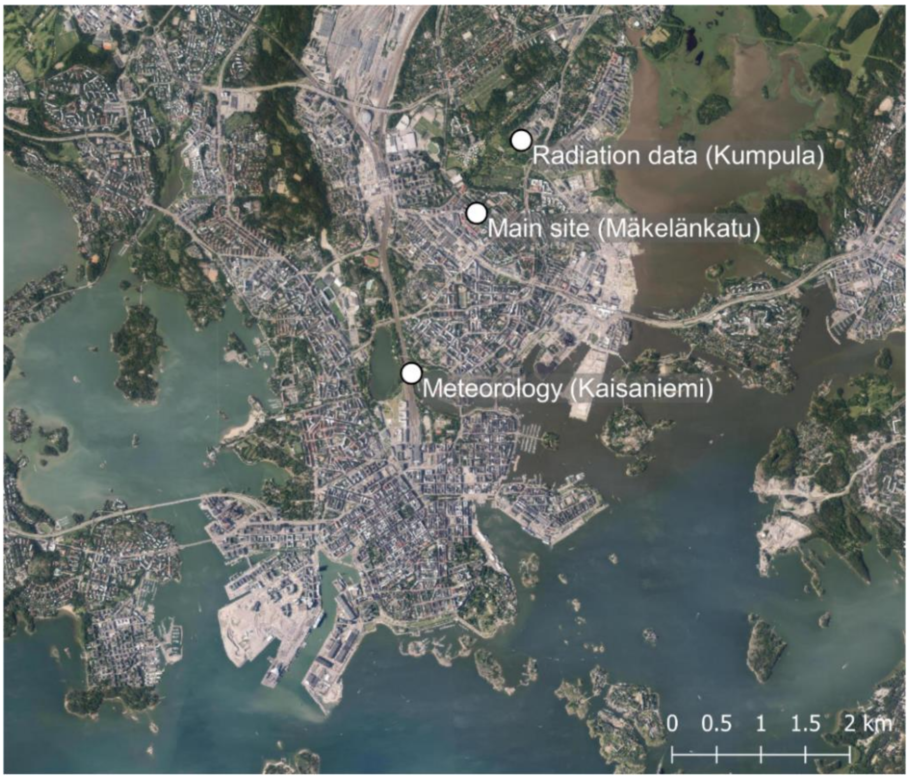

Figure S2a: A map of the city centre of Helsinki with key measurement locations. Map / aerial photograph: The City of Helsinki Map Service https://kartta.hel.fi/?setlanguage=en.

---

[1] Statistics Finland (2021) URL https://stat.fi/en/topic/population-and-society

[2] Helsinki Region Environmental Services Authority (2021) Energy and material balances and greenhouse gas emissions. URL https://www.hsy.fi/en/environmental-information/open-data/avoin-data---sivut/helsinki-region-environmental-services-authoritys-hsy-energy-and-material-balances-and-greenhouse-gas-emissions/

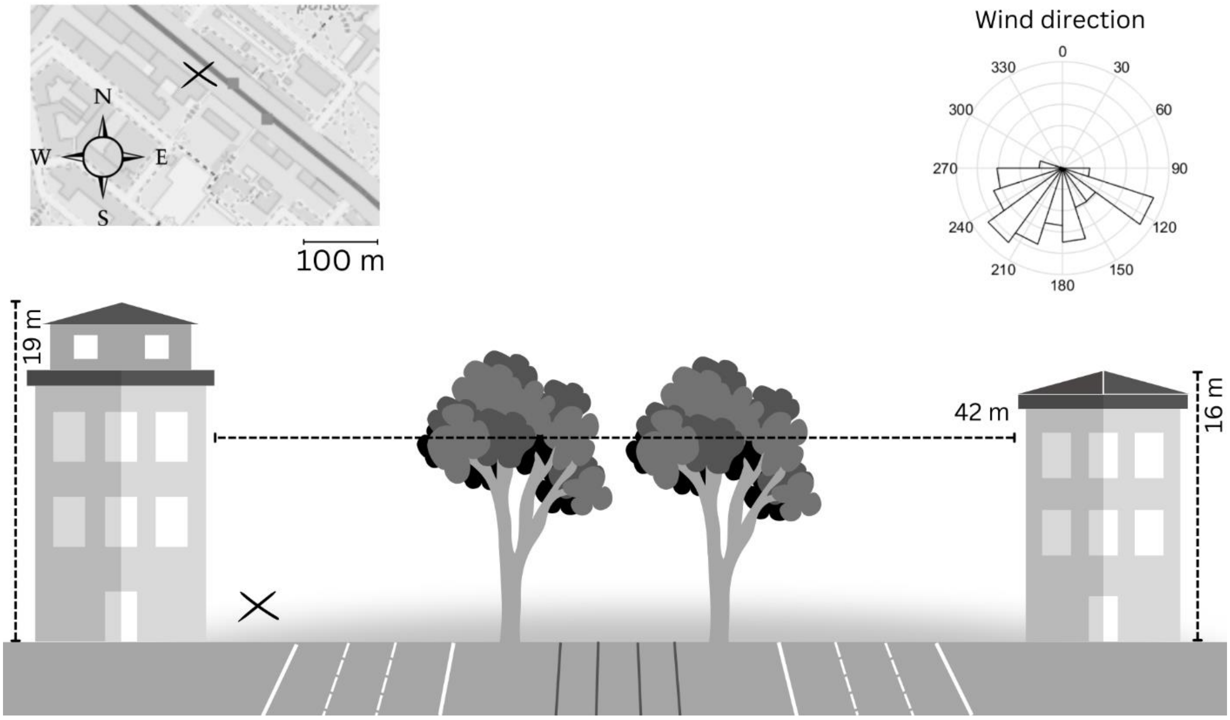

Figure S2b: The main measurement site (Mäkelänkatu): location on map, cross section of the street canyon with measurement location marked with an ‘x’, and the distribution of wind direction during the period considered in this study. Map: OpenStreetMap.

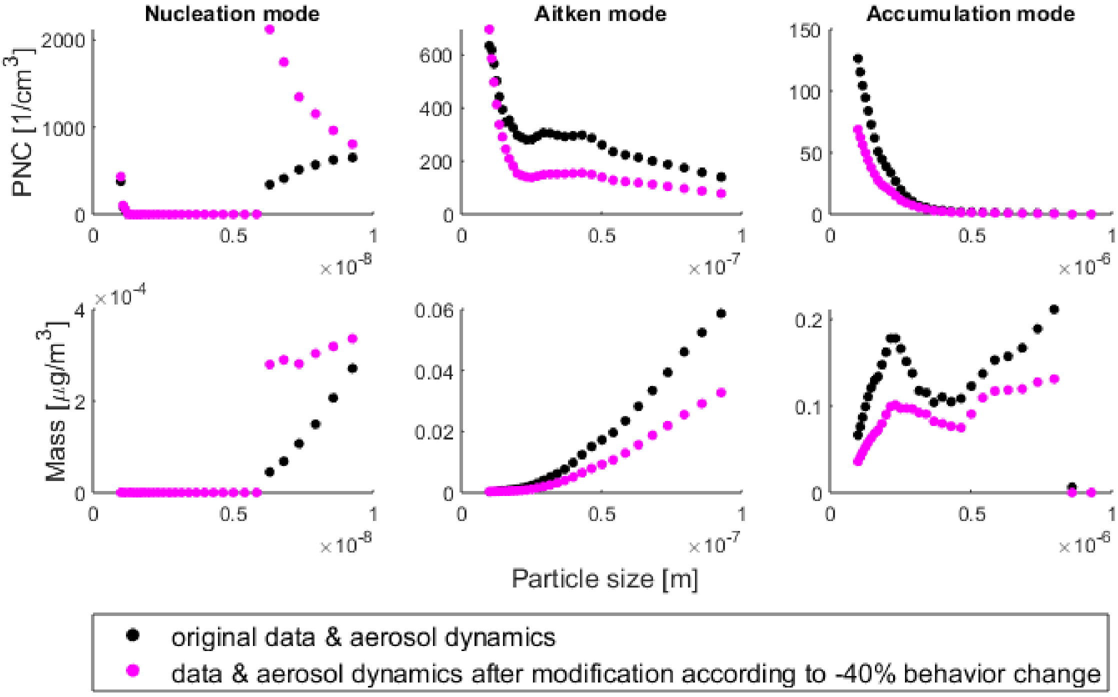

Figure S3 Particle number concentration (PNC) and particle mass concentration for different particle size modes modelled with ARCA box using the original data and the modified data (*c*=0.4). The input particle data covered sizes 6–800 nm, i.e. the measured size range without extrapolation to sub-6 nm particles. In nucleation mode, two effects can be observed: a decreased coagulation sink and enhanced cluster formation. Both effects lead to an increased PNC in nucleation mode. Compare to Figure 4 in the article.

Table S1. Comparison of modelled changes in ambient particles, when the size range of the input particle population was either 1.4–800 nm (as in the article) or 6–800 nm. Scenarios: 10%, 50% and 100% increase in pro-environmental affordances.

	Average change between model results with the original and the modified (*c* = 0.15; *c* = 0.3; *c* = 0.4) data	
Variable	Input particle data: 1.4-800 nm	Input particle data: 6-800 nm
PM_1	+2.3%; -10.0%; -16.5%	-12.1%; -18.0%; -21.4%
PNC (tot)	-12.3%; -24.6%; -32.8%	+2.5%; -2.5%; -13.9%
PNC (nuc)	-31.7%; -35.3%; -34.0%	+100.8%; +105.3%; +90.9%
PNC (UFP)	-35.0%; -35.3%; -34.0%	+4.4%; +0.18%; -11.2%
PNC (acc)	-10.0%; -20.5%; -25.6%	-19.5% -28.7; -36.5%
LDSA	-22.6%; -31.9%; -36.1%	-21.8%; -29.6%; -37.4%
